# Profiling mortality patterns and place of death in patients on maintenance hemodialysis: a retrospective study in a tertiary care academic hospital in India

**DOI:** 10.1186/s12904-025-01748-9

**Published:** 2025-04-21

**Authors:** Surya Gayathri M, Bharathi Naik, Arun Ghoshal, Anuja Damani, Shankar Prasad Nagaraju

**Affiliations:** 1 Department of Renal Replacement Therapy and Dialysis Technology, Manipal College of Health Professions, Manipal Academy of Higher Education, Manipal, Karnataka 576104 India; 2 Department of Nephrology, Father Muller College of Allied Health Science, Kankanady, Mangalore India; 3 Department of Palliative Medicine and Supportive Care, Kasturba Medical College, Manipal, Manipal Academy of Higher Education, Manipal, Karnataka 576104 India; 4 Department of Nephrology, Kasturba Medical College, Manipal, Manipal Academy of Higher Education, Manipal, Karnataka 576104 India

**Keywords:** End-stage kidney disease, Hemodialysis, Mortality patterns, Kidney supportive care, Advance care planning

## Abstract

**Background:**

End-stage kidney disease (ESKD) significantly burdens healthcare systems and increases mortality. In India, approximately 175,000 individuals are relying on maintenance hemodialysis (mHD). This study aims to analyze the place of death, mortality patterns and clinical issues surrounding death in patients with ESKD undergoing mHD at a tertiary care hospital in south India.

**Methods:**

This retrospective study reviewed the medical records of 280 patients with ESKD who underwent mHD between January 2016 and April 2022. Data were collected on demographics, financial details, comorbidities, cause of death, clinical issues, and location of death. Descriptive statistics, including means, frequencies, and proportions, were used to summarize findings.

**Results:**

Among the 280 patients on mHD, there were 98 deaths. Of these, 73.5% died in hospitals, primarily in intensive care units. Of all the patient deaths, 57.7% patients who died at home and 41.6% of hospitalized patients paid out of pocket treatment expenses. Infections and cardiovascular complications were the leading causes of death. High comorbidity and symptom burden were reported, with edema, fatigue, and breathlessness being the most common symptoms. Among the hospital deaths, withholding or withdrawing life sustaining treatments was documented in only 22.2% of cases.

**Conclusions:**

Our study provides critical insights into mortality patterns and the need for improved kidney supportive care integration in patients with ESKD. Addressing symptom burden, enhancing ACP, and implementing end of life care protocols could align healthcare delivery with patient preferences, ultimately improving the quality of end of life care in this vulnerable population. Further research is recommended to explore broader interventions and their impact on patient outcomes.

## Background

End-stage kidney disease (ESKD) is a critical stage of renal dysfunction, during which the kidney’s ability to function and maintain bodily homeostasis is severely compromised [1]. Globally, ESKD imposes a significant burden on healthcare systems and individuals, substantially affecting morbidity, mortality, and quality of life. Among the various treatment modalities available for ESKD management, hemodialysis is one of the cornerstones that provide vital renal replacement therapy to sustain life for affected individuals. A 2018 estimate put the number of patients on chronic dialysis in India at about 175,000, giving a prevalence of 129 per million population [2].

Despite the life-sustaining benefits of maintenance hemodialysis (mHD), patients with ESKD undergoing this therapy encounter multifaceted challenges, including debilitating symptoms, treatment-related burdens, and complex end-of-life (EOL) care decisions. A systematic review in 2010 estimated that approximately two-thirds of all patients with kidney failure died without receiving dialysis [3].Therefore, healthcare providers must understand the nuanced aspects of mortality patterns and the circumstances surrounding the place of death in this patient population to deliver tailored and compassionate care that aligns with patients’ preferences and values.

The landscape of EOL care for patients with ESKD undergoing mHD is complex and often characterized by a delicate balance between life-prolonging interventions and ensuring a dignified and comfortable transition toward death. Palliative care principles focusing on symptom management, psychosocial support, and shared decision-making have been increasingly recognized as integral components of care delivery for individuals with advanced kidney disease. However, the optimal integration of palliative or kidney-supportive care into the management of ESKD remains an ongoing challenge influenced by various factors, such as healthcare infrastructure, cultural norms, and provider attitudes [4].

The monthly cost of mHD in most private hospitals in India is 12,000 INR (Indian Rupee), which represents an annual cost of 1.4 million INR, equivalent to 3000 USD (US Dollars), which is in sharp contrast to an annual fee of 60,000 USD in the United States and the United Kingdom. Although the cost of mHD is lower in India than in most other countries, more than 90% of Indians cannot afford it [5]. The mortality patterns of patients with ESKD on mHD reflect a diverse array of contributing factors, including age, comorbid conditions, dialysis adequacy, and access to healthcare resources. Cardiovascular events, infections, and complications related to comorbidities are among the leading causes of death in this population [6]. Furthermore, the place of death has a profound significance for patients and their families, with implications for care quality, emotional well-being, and resource use. While hospital deaths are prevalent, many patients express a preference for dying at home, highlighting the importance of aligning care delivery with individual preferences and values [7].

Against this background, this study aimed to comprehensively analyze the mortality patterns and places of death among patients with ESKD who underwent mHD at a single centre in South India.

## Methods

### Study design and population

This retrospective study analyzed the hospital medical records of patients with ESKD who underwent mHD at Kasturba Medical College and Hospital in Manipal, India, between January 1, 2016, and April 30, 2022. Patients aged 18 years or older who were on mHD and died during the study period were included in the study. Individuals who were undergoing acute dialysis (duration of < 3 months), post-kidney transplant recipients, or patients who had experienced transplant failure were excluded from the study. Following discharge from the hospital, patients who had discontinued dialysis or those discharged against medical advice (DAMA) were contacted by the hospital staff to verify their status, specifically their death. The symptom parameters were evaluated using the Integrated Palliative Care Outcome Scale (IPOS). We assessed the symptoms as fair when there were occasional or mild, single incidents and if the patient was not bothered by the symptoms. Moderate to severe, continuous, and overwhelming symptoms were considered poor. The symptoms and cause of death were collected from the hospital’s medical records and patient’s death certificate for the patients who died in hospital settings.

### Data collection

We extracted the demographic characteristics (age, gender, healthcare financing), comorbidities, dialysis vintage (duration of mHD), frequency of dialysis, hospitalizations in the last year of life, cause of death, place of death, and care setting-specific factors (e.g., symptom burden, use of breathing supports and EOL care documentation) from the hospital medical records. All patients were included in the analysis.

### Statistical analysis

We cleaned the data by addressing missing values, outliers, and inconsistencies. All statistical analyses were performed using Python. Data were expressed as mean ± standard deviation (SD) for continuous variables and as frequencies (percentages) for categorical variables. The normality of data distribution was assessed using the Shapiro-Wilk test (or Kolmogorov-Smirnov test, if applicable). Student’s t-test was used for normally distributed continuous variables for comparisons between two independent groups, while the Mann-Whitney U test was applied for non-normally distributed data. Categorical variables were compared using the Chi-square test (χ² test) or Fisher’s exact test, as appropriate. A *p*-value < 0.05 was considered statistically significant.

## Results

Medical records of 280 patients undergoing mHD were reviewed during the study period. Among these, 98 patients succumbed, and their records were included in the analysis. The demographics, insurance status, and dialysis-related details of all patients are presented in Table 1. The average age of the patients was 63 ± 14 years (range, 18–85 years). Among the included patients, 73 (74%) were men, 92 (94%) were married, and(69%) were employed.

**Table 1:** Demographics, insurance status, and dialysis-related details of all patients (*n* = 98)

Items		Hospital deaths (*n* = 72)				Home deaths (*n* = 26)	*p*-value (Hospital vs. Home deaths)
		Inpatient ward *n* = 11(%)	Intensive care unit *n* = 53(%)	Discharge against medical advice *n* = 8(%)	Total *n*= (72%)		
Average age(years)		63	62	55	62	58	0.27
Gender	Male	9(81.8)	41(77.3)	1(12.5)	51(70.8)	22(84.6)	0.26
	Female	2(18.1)	12(22.6)	7(87.5)	21(29.2)	4(15.4)	
Marital status	Single	0(0)	2(3.8)	3(37.5)	5(6.9)	1(3.8)	0.93
	Married	11(100)	51(96.2)	5(62.5)	67(93.0)	25(96.1)	
Employment	Employed	7(63.6)	40(75.5)	2(25.0)	49(68)	19(73.1)	0.82
	Unemployed	4(36.3)	13(24.5)	6(75.0)	23(31.9)	7(26.9)	
*Comorbidities	Cardiovascular	5(45.4)	19(35.8)	3(37.5)	27(37.5)	12(46.1)	
	Others	6(54.5)	41(77.3)	6(75.0)	53(73.6)	11(42.3)	
	Pulmonary	4(36.4)	27(50.9)	4(50.0)	35(48.6)	7(26.9)	Not applicable
	Liver disease	0(0)	3(5.6)	0(0)	3(4.2)	2(7.7)	
	Diabetes mellitus	6(54.5)	32(60.4)	4(50)	42(58.3)	2(7.7)	
	Hypertension	11(100)	50(94.3)	8(100)	69(95.8)	24(92.3)	
Dialysis vintage	1–3 years	2(18.2)	17(32.1)	2(25)	72(29.2)	19(73.1)	**0.00024**
	4–16 years	9(81.8)	36(67.9)	6(75.0)	51(70.8)	7(26.9)	
Number of dialysis cycles per week	Twice weekly	9(81.8)	44(83.0)	5(62.5)	58(80.5)	22(84.6)	0.87
	Thrice weekly	2(18.2)	9(16.9)	3(37.5)	14(19.4)	4(15.4)	
Financial support	Insured/ Partially insured	5(45.5)	33(62.3)	4(50)	42(58.3)	11(42.3)	0.240
	Not insured	6(54.5)	20(37.7)	4(50.0)	30(41.7)	15(57.7)	

* There may be multiple comorbidities in a single patient

Regarding dialysis frequency, 80 (81.6%) individuals underwent dialysis twice weekly, whereas the remaining were on thrice-weekly sessions. Of those receiving dialysis, 58 (59.2%) had been on mHD for more than 4 years. The most prevalent comorbidity was hypertension in 93 (94.9%) patients, followed by diabetes in 57 (58.2%) patients, chronic lung disease in 42 (42.9%) patients, and cardiovascular disease in 39 (39.8%) patients.

Of the 98 patients, 26 (26.5%) died at home, and 72 were hospitalized. For hospitalized patients, 8 (11.1%) were discharged against medical advice (DAMA). Of the remaining, 53 (73.5%) died in the Intensive Care Unit (ICU) and 11 (15.3%) died in the ward.

In terms of health insurance, 42 (58.3%) hospitalized patients were insured (25 fully insured, 17 partly insured), whereas 30 (41.6%) patients paid out of pocket. Of those who died at home, 15 (57.7%) did not have insurance. For the subgroup of people who had no insurance coverage (*n* = 45), 26 (57.8%) were hospital deaths, 15 (33.3%) were home deaths, and 4(8.9%) patients were discharged against medical advice (DAMA). In a subset analysis of patients with no insurance in any setting, there was a higher number of uninsured men (35, 77.8%) than women (10, 22.2%), more uninsured individuals were married (41, 91.1%) than unmarried (4, 8.9%), and 14 (31.1%) had no occupation listed. Among the in-hospital deaths, the proportion of uninsured patients who signed ‘Do not resuscitate’ (DNR) was 8 out of 26 (30.8%), whereas 8 out of 38 (21%) were insured patients.

The most common cause of death was infection and sepsis, accounting for 63 (87.5%) cases. Cardiac and vascular complications were the second most common immediate causes, occurring in 40 (56.3%) patients. These statistics were calculated according to the mortality of 64 patients who died in hospital settings.

The mortality pattern analysis further revealed other factors, such as the location of death, mode of breathing support, EOL care planning, and number of hospital admissions in the last year of life (Table 2).

**Table 2:** End-of-life symptoms, supports, and care preferences of all hospitalized patients (*n* = 72)

	Items	Inpatient ward *n* = 11(%)	Intensive care unit *n* = 53(%)	Discharge against medical advice *n* = 8(%)
Withholding/ withdrawal of artificial life supports	Documented	4 (36.3)	12(22.6)	0(0)
More than a week spent as an inpatient		3(27.3)	20(37.7)	5(62.5)
On respiratory support	Ventilator	5(45.4)	42(79.2)	0(0)
	Non-invasive ventilation	0(0)	6(11.3)	6(75.0)
Symptom Management	Fair	9(81.8)	40(75.5)	5(62.5)
	Poor	2(18.2)	13(24.5)	3(37.5)
More than 4 hospital admission in last 1 year		2(18.2)	13(24.5)	4(50.0)

Notably, 53(73.6%) patients died in the ICU, of which 47(88.6%) required mechanical ventilation. Among the patients analyzed, 19 (26.4%) experienced multiple hospitalizations during the final year of life. Additionally, all patients required physical assistance for activities of daily living. The EOL care directive for withholding or withdrawal of artificial life-sustaining treatments was documented in only 16 (22.2%) patients, of whom 4 died in the ward setting, and 12 were in the ICU setting. All hospitalized patients had a high symptom burden, with the most common symptoms being edema, fatigue, and breathlessness. Of all patients who were DAMA (*n* = 8), none had documented advance care planning (ACP), and most had a high symptom burden at the EOL.

Implementing the EOL directive [8] or withholding or withdrawing artificial life-sustaining treatments in 16 patients. This included 11 patients who withdrew from dialysis and 5 patients who withheld dialysis while also escalating other artificial life-sustaining treatments, such as mechanical ventilation, inotrope support, and cardiopulmonary resuscitation. In all these patients, ACP discussions were initiated near death. Due to the absence of hospital care-seeking among home-deceased patients, the specifics of their EOL phase remain unknown. It was interesting that home and hospital deaths were comparable in terms of demographics, marital (*p* = 0.93), employment (*p* = 0.82), insurance (*p* = 0.24) status, and dialysis frequency (*p* = 0.87). However, there was a significant difference in dialysis vintage between home and hospital deaths (*p* < 0.001). A higher proportion of home deaths had a shorter dialysis duration (1–3 years), whereas a higher proportion of hospital deaths had a longer dialysis duration (> 4 years).

## Discussion

The findings of this study provide valuable insights into the mortality patterns, demographics, comorbidities, and EOL of patients undergoing hemodialysis. Comparing these results with the existing literature sheds light on several important aspects. The study revealed that a significant proportion of hemodialysis patients die in hospital settings, more so in ICUs. This finding aligns with previous research indicating that hemodialysis patients have a higher likelihood of hospitalization and experience a substantial burden of EOL care interventions, including emergency department visits and intensive care unit admissions [9]. The demographic profile, comorbidity scores, and mortality patterns, including the symptom profile and cause of death, of the mHD patients in this study corresponded with the findings in the literature [10–13]. These individuals have complex healthcare needs and challenges that impact their prognosis and quality of life. These findings underscore the importance of infection prevention strategies, proactive management of comorbidities, cardiovascular risk factors, and symptoms for improving outcomes in this vulnerable population [14]. Additionally, implementing EOL care planning, as evidenced by the BLUE MAPLE document, reflects a proactive approach to addressing EOL preferences and ensuring patient- and family-centered care [15]. This highlights the importance of timely and appropriate integration of kidney supportive care, symptom management, communication, preference discussion, and ACP in patients with ESKD [12]. These interventions are essential to alleviate suffering and improve the quality of life of hemodialysis patients nearing the EOL. In India, out-of-pocket healthcare expenses are high. ACP discussions could help reduce the undue financial burden on these patients and their families. Such individuals may be more open to discussions about EOL care preferences for withholding or withdrawal from dialysis and other artificial life-sustaining treatments like mechanical ventilation, cardiopulmonary resuscitation, inotrope supports, or ICU admissions.

Although no definitive associations were found due to the small sample size, certain mortality patterns were observed in patients with ESKD across different settings. In hospital wards, the severity of comorbidities and complications related to hemodialysis, such as infections and electrolyte imbalances, contribute to higher mortality risks. Multiple organ dysfunction syndrome (MODS), septic shock, and severe infections are the primary triggers of death in patients with ESKD in the ICU. The need for ongoing dialysis complicates the outcome. Patients with DAMA may experience increased mortality risks due to a lack of follow-up care, financial constraints, or a shift in focus from life-prolonging measures to quality of life, leading to unmanaged complications and worsened outcomes. Such patterns have been observed in literature from India [16–19]. and the rest of the world [20]. Although our study observed that patients with a shorter duration of dialysis were more likely to die at home, there is a lack of existing literature directly comparing dialysis vintage with the place of end-of-life care. However, prior studies have indicated that a longer dialysis vintage is associated with increased complications and a higher frequency of hospitalizations [21, 22]. These clinical trajectories may, in turn, influence end-of-life care preferences and decision-making. It is important to note, however, that such decisions are shaped by a complex interplay of factors related to the patient, family, disease status, and healthcare providers [23–25].

Limitations: The study included patients on hemodialysis in a specific geographic area or healthcare setting, which may have led to selection bias. There may have been limitations in data collection, such as incomplete medical records or reliance on self-reported information, which may have affected the accuracy of the findings. The retrospective study design might have led to the missing important details about EOL care, symptom burden, patient’s and family perspectives about EOL care, and financial implications experienced by hemodialysis patients. We lacked sufficient data on home deaths to draw meaningful conclusions.

### Study strengths

This study will provide insights to enhance our kidney-supportive care services provision in terms of a better understanding of patterns of mortality, the timing of advance care planning, eliciting and documenting patient and family preferences, and thus improving patient and family satisfaction. This study contributes to our understanding of the challenging choices faced by patients with ESKD regarding aggressive treatment options, such as dialysis and other artificial life supports, vs. comfort-focused care. The findings from this research help the medical team and family members discuss the illness trajectory and what the patient may want in different situations, which can reduce family conflict and the burden of decision-making during times of crisis.

### Suggestions for future research

Conducting a multicenter or nationwide study to capture a more diverse representation of hemodialysis patients and their EOL experiences would provide a more comprehensive understanding of the patterns observed in this study. Prospective studies that involve real-time data collection and follow-up could offer more detailed insights into the symptom burden, financial implications, and decision-making processes of hemodialysis patients at the EOL. It may be possible to better tailor interventions and policies to specific needs and contexts by investigating cultural, societal, and regional variations in EOL care and financial implications for hemodialysis patients. Investigating the impact of specific interventions, such as ACP programs or insurance coverage, on the EOL outcomes of high risk hemodialysis patients would provide valuable information for improving care delivery and support systems in this population.

## Conclusion

This study highlights the complex interplay of demographic characteristics, comorbidities, and healthcare access on mortality outcomes in patients undergoing mHD. Despite similar demographic and clinical characteristics between home and hospital deaths, most patients who underwent dialysis for > 4 years died in the hospital, often requiring aggressive management in the ICU in the form of artificial life-sustaining treatments. The high symptom burden and lack of ACP among patients underscore the need for improved EOL care protocols. The timely implementation of advance care directives can facilitate more compassionate and informed decision-making in such challenging situations.

## Data Availability

Available with the corresponding author on request.

## Abbreviations

ACP: Advance Care Planning
DAMA: Discharge Against Medical Advice
ESKD: End-Stage Kidney Disease
mHD: Maintenance Hemodialysis
MODS: Multiorgan Dysfunction Syndrome
EOL: End of Life
